# *In-Vivo* Imaging of Cell Migration Using Contrast Enhanced MRI and SVM Based Post-Processing

**DOI:** 10.1371/journal.pone.0140548

**Published:** 2015-12-14

**Authors:** Christian Weis, Andreas Hess, Lubos Budinsky, Ben Fabry

**Affiliations:** 1 Biophysics Group, University of Erlangen-Nuremberg, Erlangen, Germany; 2 Clinic of Diagnostic and Interventional Radiology, University Hospital Heidelberg, Heidelberg, Germany; 3 Pharmacology, University of Erlangen-Nuremberg, Erlangen, Germany; City of Hope, UNITED STATES

## Abstract

The migration of cells within a living organism can be observed with magnetic resonance imaging (MRI) in combination with iron oxide nanoparticles as an intracellular contrast agent. This method, however, suffers from low sensitivity and specificty. Here, we developed a quantitative non-invasive *in-vivo* cell localization method using contrast enhanced multiparametric MRI and support vector machines (SVM) based post-processing. Imaging phantoms consisting of agarose with compartments containing different concentrations of cancer cells labeled with iron oxide nanoparticles were used to train and evaluate the SVM for cell localization. From the magnitude and phase data acquired with a series of ${\text{T}}_{2}^{*}$-weighted gradient-echo scans at different echo-times, we extracted features that are characteristic for the presence of superparamagnetic nanoparticles, in particular hyper- and hypointensities, relaxation rates, short-range phase perturbations, and perturbation dynamics. High detection quality was achieved by SVM analysis of the multiparametric feature-space. The *in-vivo* applicability was validated in animal studies. The SVM detected the presence of iron oxide nanoparticles in the imaging phantoms with high specificity and sensitivity with a detection limit of 30 labeled cells per mm^3^, corresponding to 19 μM of iron oxide. As proof-of-concept, we applied the method to follow the migration of labeled cancer cells injected in rats. The combination of iron oxide labeled cells, multiparametric MRI and a SVM based post processing provides high spatial resolution, specificity, and sensitivity, and is therefore suitable for non-invasive *in-vivo* cell detection and cell migration studies over prolonged time periods.

## Introduction

Histological studies of cell migration in animal models require sacrificing the animals. Therefore, the data obtained from any given animal is limited to a single point in time. For certain processes such as the formation of metastases, regional tumor growth and micrometastatic progression, the colonization of biomaterials with cells, or the migration of stem cells, it is essential to observe the distribution pattern of injected cells in the same animal at multiple time points. Non-invasive imaging techniques such as optical imaging (OI), computed tomography (CT) or conventional magnetic resonance imaging (MRI) have the potential to circumvent this problem [1]. Limitations of OI-based cell tracking techniques include limited depth of penetration, limited quantification and poor spatial resolution due to photon scatter [2]. In comparison, CT, and MRI allow for tracking of cell position at any tissue depth at the expense of some detail, sensitivity, and specificity [3].

MRI is an imaging modality with superior soft-tissue-contrast, but cannot resolve individual cells. To distinguish between the cells of interest and the animal’s background tissue, and therefore to increase the sensitivity and specificity of MRI, it has been suggested to label cells with superparamagnetic iron oxide (SPIO) contrast agents prior to injection [4]. Tumor cell migration, regional tumor growth and micrometastatic progression could be investigated by labeling *in-vitro* cultures of metastatic tumor cells with iron oxide particles, injecting these cells into an animal, and tracking them over time with MRI. This strategy has been applied to monitor iron oxide labeled NSC-derived oligodendroglial progenitors within the rat brain [5], to detect labeled metastatic melanoma cells within the mouse lymph nodes [6], and more recently to observe the migration of dendritic cells into the drain lymph nodes of mice [7]. However, these techniques are limited in terms of the smallest detectable cell accumulation and the unambiguous identification of superparamagnetic nanoparticles [8]. Previous studied showed a limit of ∼125 cells/voxel for unambiguous detection of iron oxide *in-vitro* [9].

In the current study, an accurate cell localization method with high specificity and sensitivity for SPIO labeled cells is presented. The method employs multiparametric magnetic resonance imaging in combination with support vector machine (SVM)-based data postprocessing to follow the migration of any cell type anywhere in the animal except in the lungs. For a proof-of-principle, we label cancer cells with superparamagnetic iron oxide particles and localize them in agarose phantoms. Moreover, in an *in-vivo* rat study we confirm the sensitivity and specificity of the method for localizing labeled cells at the whole body level.

## Results

### *In-vitro* studies

**Fig 1:**
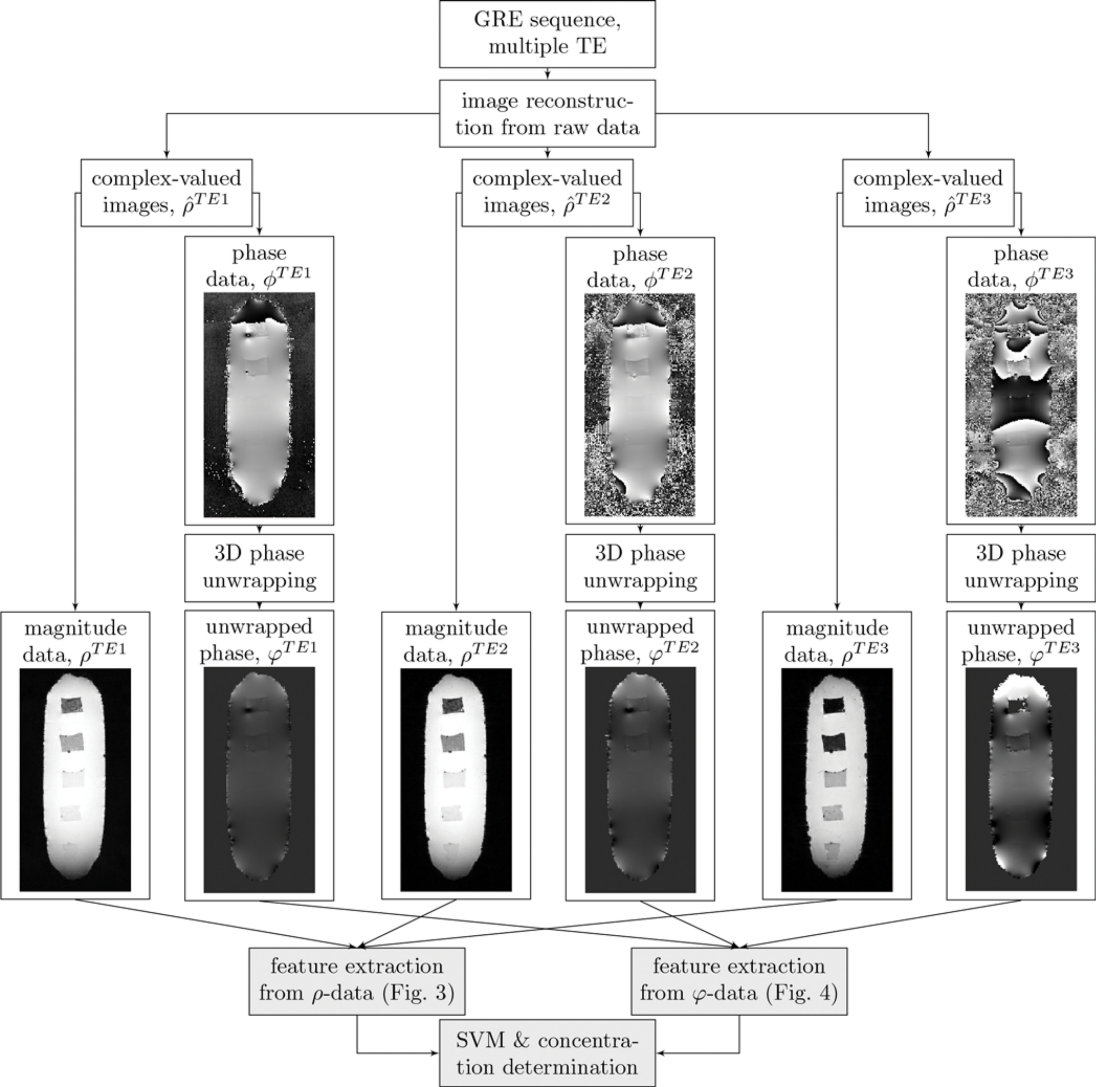
Data flow of the localization algorithm. Magnitude (*ρ*-data) and phase (*φ*-data) are reconstructed from the raw data, afterwards features characteristic for the presence of iron oxide particles are extracted and analyzed by the support vector machine (SVM). This figure shows the data flow for an agarose block phantom.

**Fig 2:**
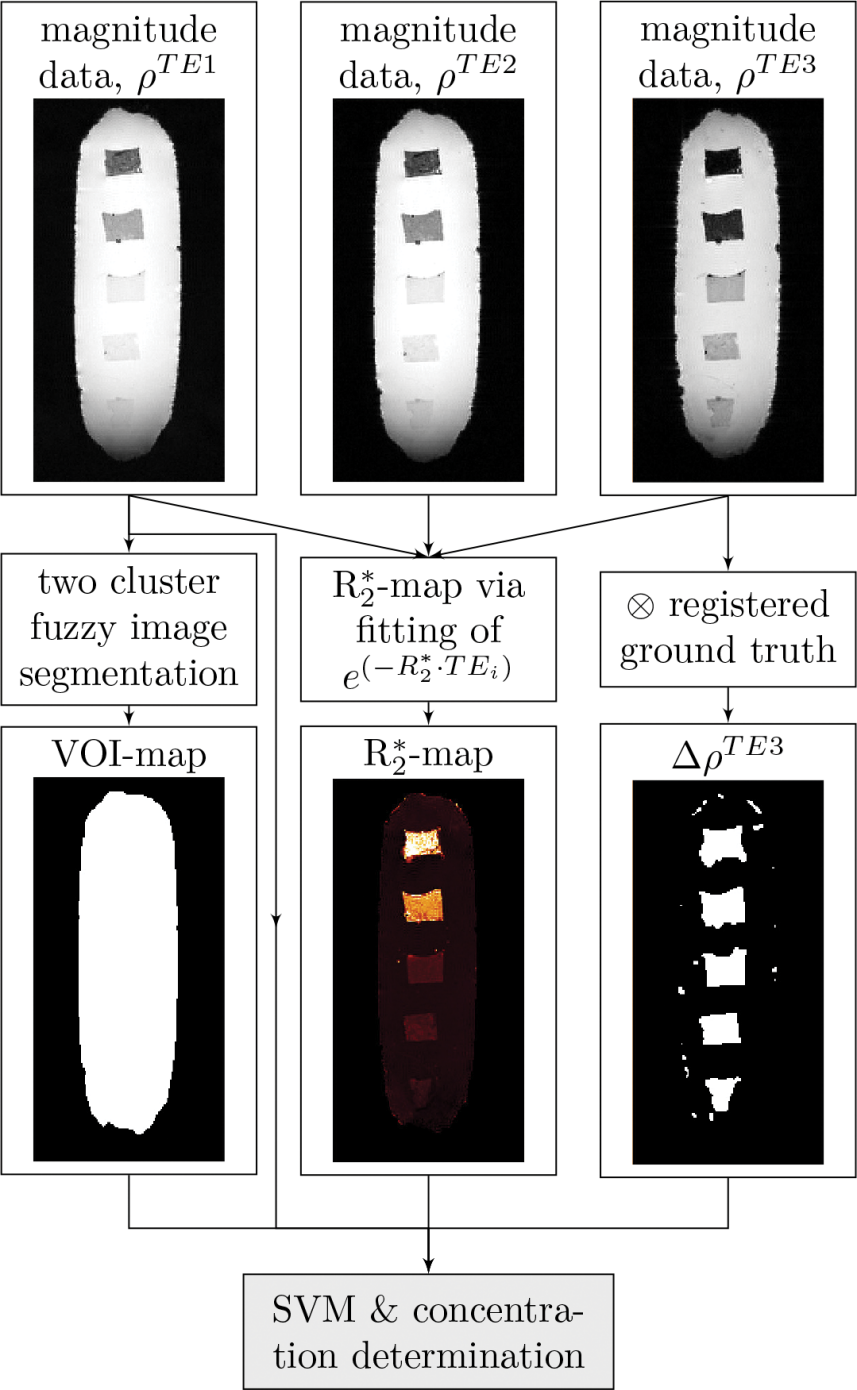
Overview of the feature extraction workflow from magnitude (*ρ*-data).

**Fig 3:**
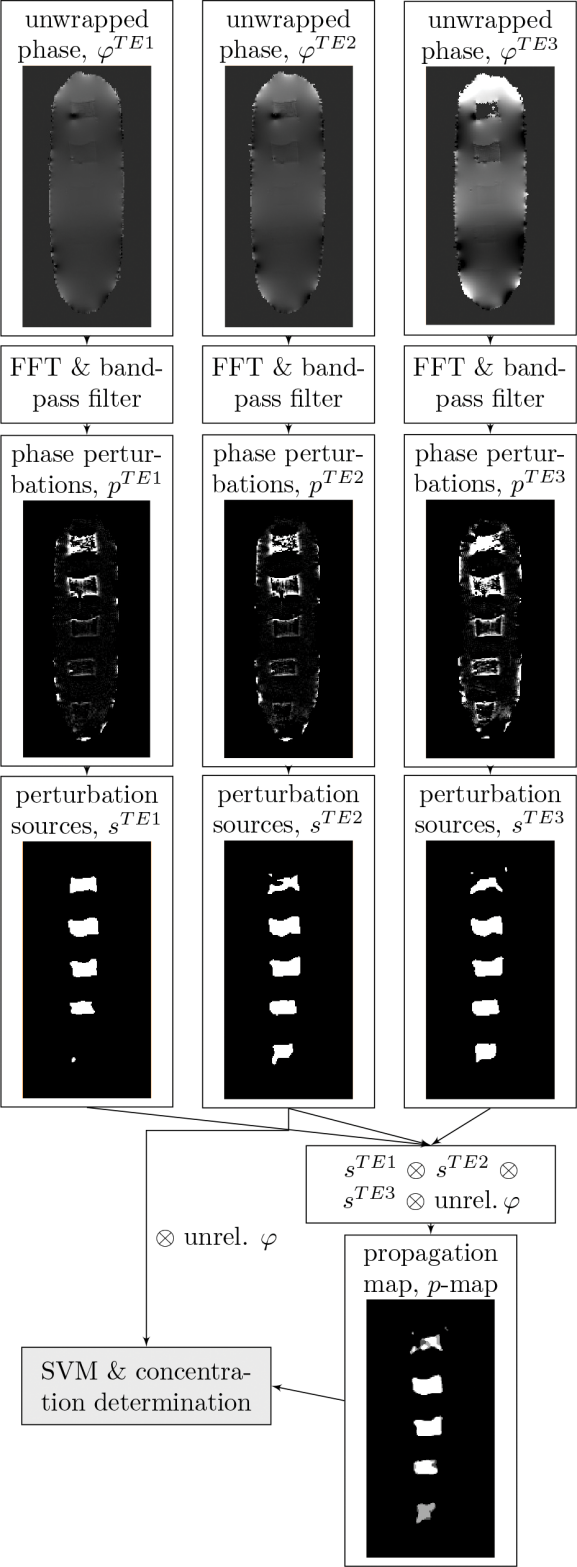
Overview of the feature extraction workflow from phase (*φ*-data).

**Fig 4:**
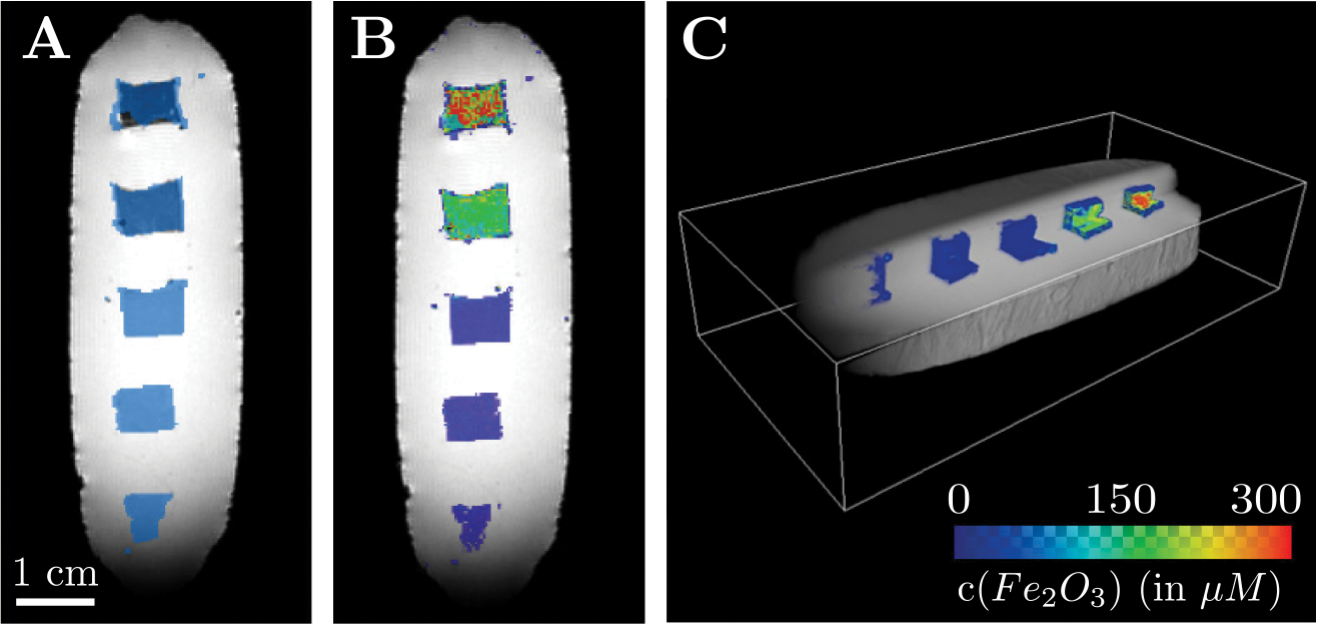
Overlay of the MRI magnitude data (grayscale) and the results of the SVM-based localization method for an agarose block phantom (color-coded). A: Result of the C-SVC in the medial plane of the block phantom. Voxels classified as *containing labeled cells* are colored in blue. B: Corresponding iron oxide concentration map. C: 3D concentration map with corner-cut. The colorbar applies to B and C.

In a first step, the machine learning-based localization algorithm (Fig 1) was trained and applied on agarose block phantoms containing multiple subvolumes of iron oxide nanoparticles at different concentrations. Features characteristic for the presence of iron oxide particles were then extracted from magnitude (Fig 2) and phase data (Fig 3). Applying the SVM-model on these features gives a 3D map in which each voxel is classified as either *containing iron oxide* and *not containing iron oxide* (Fig 4A). Finally, an iron oxide concentration map is calculated from the ${\text{R}}_{2}^{*}$-map of the iron oxide containing voxels (Fig 4B and 4C).

#### Training and evaluation of the SVM

**Fig 5:**
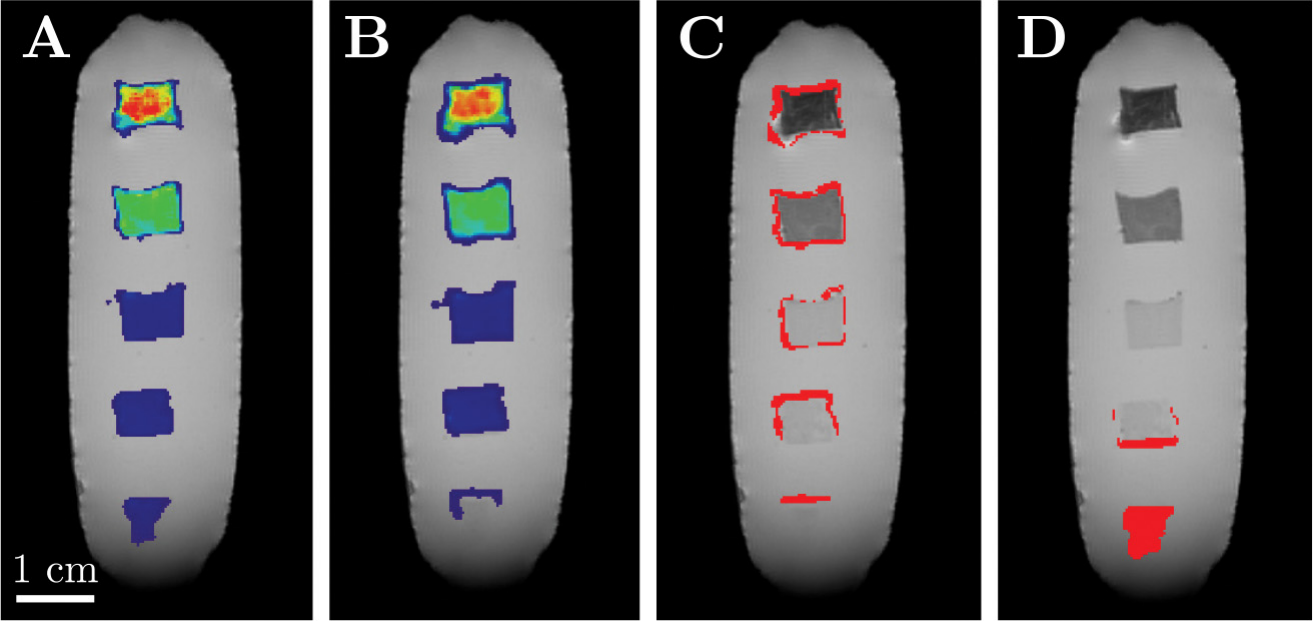
Overlay of the MRI magnitude data (grayscale) and SVM results (color-coded). A: Iron oxide concentration map for the training phantom. B: Iron oxide concentration map for the test phantom. The image was registered to the training phantom. C: *False positive* voxels ($\hat{=}$ voxels that are falsely classified as *containing labeled cells*) in the test phantom (data from B). D: *False negative* voxels ($\hat{=}$ voxels that are falsely classified as *not containing labeled cells*) in the test phantom (data from B).

We compared the SVM classification results of the training phantom and another agarose phantom that was not used for SVM training but only for SVM evaluation. In the training phantom, the algorithm correctly identified all subvolumes with different iron oxide concentrations (Fig 5A). The mean ratio of detected volume (vol_*detected*_) to real inlay volume known from the phantom fabrication process (vol_*real*_) was 1.00 ± 0.03 (Table 1, mean ± sem from 1 training phantom, 3 scans).

**Table 1:** Comparison of the the calculated iron oxide concentrations and volumes for the training and evaluation phantoms.

*c*_*r*_(*Fe*_2_ *O*_3_) in *μ*M	*c*_*r*_(cells) in cells/mm^3^	phantom type	vol_*d*_/vol_*r*_	*c*_*d*_(*Fe*_2_ *O*_3_) ± 1 ⋅ *σ* in *μ*M
19	30	evaluation	0.40	14.05 ± 8.25
		training	0.97	23.41 ± 5.07
38	60	evaluation	1.03	36.96 ± 10.66
		training	0.98	39.32 ± 5.12
78	120	evaluation	1.10	67.15 ± 3.19
		training	1.04	66.29 ± 12.75
156	240	evaluation	1.12	165.31 ± 31.75
		training	1.03	157.35 ± 20.03
313	480	evaluation	1.04	254.34 ± 52.10
		training	0.99	283.19 ± 56.26

Comparison of the detected iron oxide volumes (vol_*d*_) and concentrations (c_*d*_) with the real volumes (v_*r*_) and concentrations (c_*r*_) used during phantom fabrication. The comparison was carried out on the training phantom of the SVM and an evaluation phantom.

For the evaluation phantom, the algorithm gave a correct voxel classification for all inlays except the inlay with the lowest concentration (19 μM iron oxide ≈ 2 labeled cells per voxel) where parts of the inlay volume were not detected entirely (Fig 5B). The average v_*detected*_/v_*real*_-ratio for the evaluation phantom was of 0.94 ± 0.30 (Table 1, mean ± sem from 2 evaluation phantoms, 3 scans). Excluding the inlay with the lowest concentration gave an average v_*detected*_/v_*real*_-ratio of 1.07 ± 0.04. This indicates that the volumes were slightly overestimated. A small ‘halo’ of *false positive* voxels around areas with larger iron oxide concentration was visible, confirming this overestimation.

#### Sensitivity and specificity

To analyze the sensitivity and specificity of the SVM, we quantified the voxels’ classification results in the evaluation phantom as *true positives*, *false positives*, *true negatives*, and *false negatives*. *False positives* were always found as a ‘halo’ around the nanoparticle-containing inlays and not as scattered voxels throughout the phantom (Fig 5C). *False negatives* were only found in the inlay with the lowest concentration of labeled cells (Fig 5D), and here only near the edge of the phantom (Fig 5B). This suggests that the *false negative* voxels were not per se a result of low signal but rather were caused by the combination of low signal, deviation from the linear regime near the edge of the gradient coil, and imperfect image registration.

Although the SVM’s sensitivity for low SPIO concentrations seems to be lower when the model is applied on the evaluation dataset, the model’s specificity is the same (except for the lowest iron oxide concentration). The SVM’s mean specificity for the evaluation dataset was 0.95 ± 0.06 (mean ± std of five inlays of labeled cells), with a higher specificity for the inlays with a low SPIO concentration (Table 2). The SVM’s mean sensitivity for the evaluation dataset was 0.83 ± 0.26 (mean ± std of five inlays of labeled cells) (Table 2). Excluding the sensitivity for the lowest concentration of labeled cells (19 μM), gave a mean sensitivity of 0.95 ± 0.05 (mean ± std).

**Table 2:** Sensitivity and specificity of the SVM for the evaluation phantom.

*c*_*real*_(*Fe*_2_ *O*_3_) in *μ*M	Sensitivity (true pos./real pos.)	Specificity (true neg./real neg.)
19	0.38	1.00
38	0.88	0.95
78	0.95	0.95
156	0.96	0.98
313	0.99	0.85

Sensitivity and specificity of the SVM for the evaluation phantom. Sensitivity was calculated by dividing the number of *true positives* by the number of real positives. Specificity was calculated by dividing the number of *true negatives* by the number of real negatives. The sensitivity increased with the concentration of labeled cells in agarose gel. The specificity decreased with increasing concentration of labeled cells.

#### Detection limit

In previous work, the ${\text{R}}_{2}^{*}$ detection limit was reported as 70 cells per mm^3^ for a uniform agarose background [10], and the unambiguous identification of iron oxide nanoparticles was not possible [11] in an environment with a non-uniform ${\text{R}}_{2}^{*}$ background. In contrast, our new localization method relies on multiple features characteristic for the presence of iron oxide and thus provides a lower detection limit of 30 cells per mm^3^ (Figs 4 and 5).

#### Concentration estimation

From the ${\text{R}}_{2}^{*}$ values of the voxels classified as containing iron oxide, we calculated the iron oxide concentration, based on relaxation rate measurements performed on an iron oxide nanoparticle dilution series. Estimated values were accurate within 10% on average. Voxels with the highest concentration, however, were underestimated by 19% on average (Table 1), because the signal intensity at the longest TE (11.34 ms) was too low, and therefore too close to the background signal intensity, for a reliable exponential fit for computing R2*.

### *In-vivo* studies

**Fig 6:**
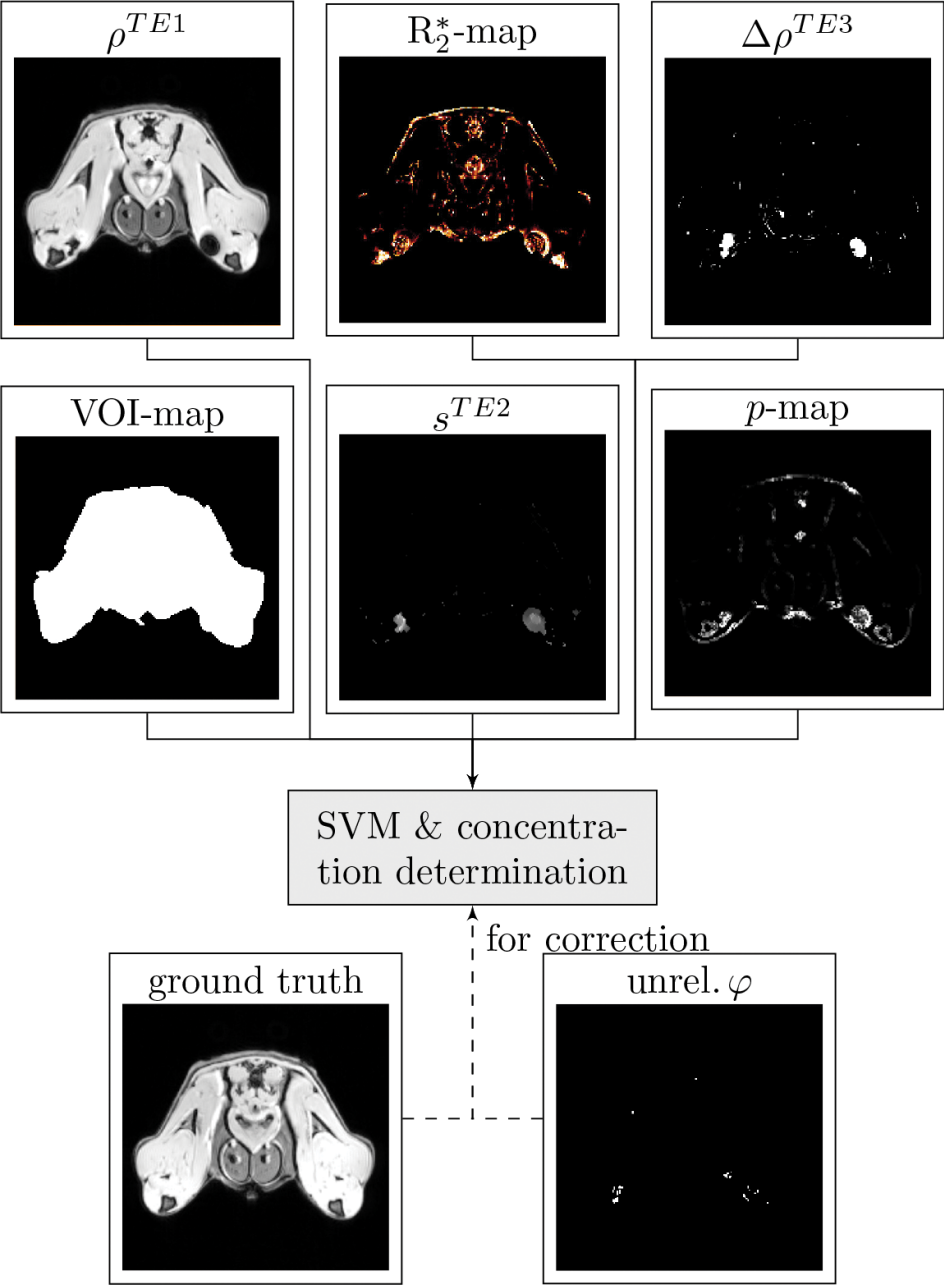
Overview of the features extracted from the image data of the *in-vivo* study.

To evaluate the method’s specificity and sensitivity *in-vivo*, two different concentrations of iron oxide labeled cells were injected the rear limbs of a rat (500,000 CC531 cells labeled with 30 μg iron oxide were injected the left limb, 500,000 R1H cells labeled with 50 μg of nanoparticles were injected in the right limb). The baseline MRI scan was acquired before injection, the second scan was performed 24 h after administration of labeled cells. The classification features described above were extracted (Fig 6), and the *in-vitro* trained SVM-model was used for classification of the rat image data.

**Fig 7:**
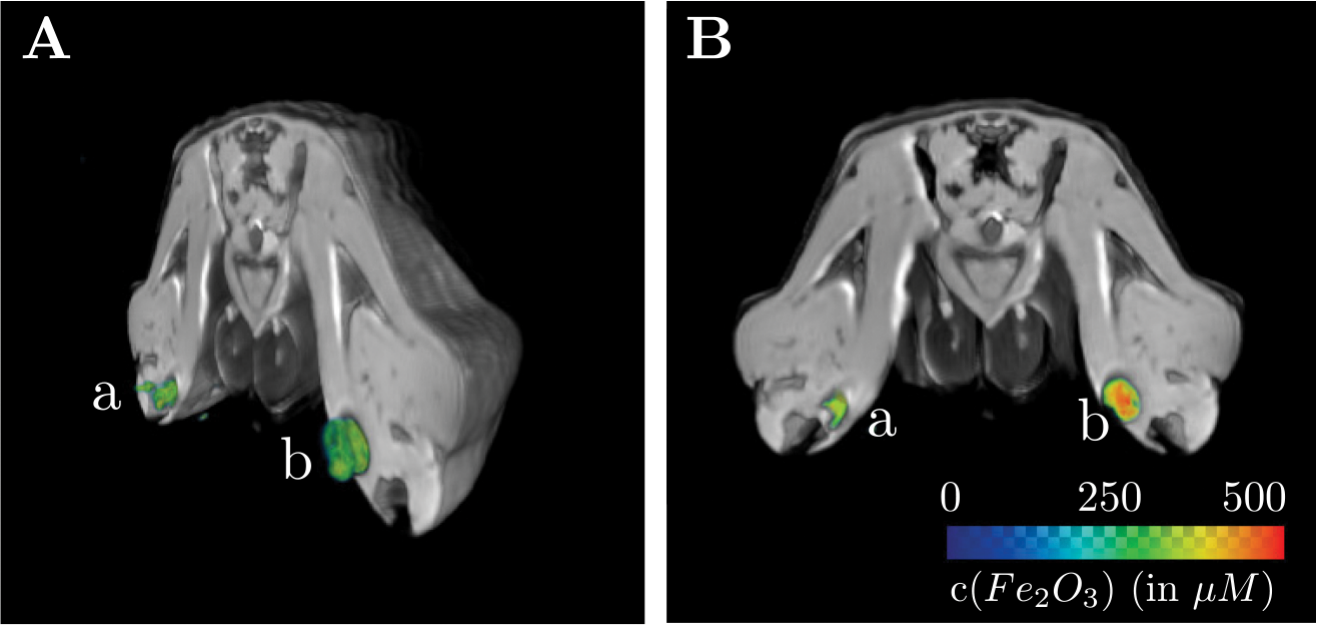
Results of the SVM-based localization method for the *in-vivo* study. A: Cross-section of a 3D reconstruction of the rat’s pelvis together with a rendering of the total volume of identified cancer cell aggregates. 500.000 CC531 cells labeled with 30 *μ*g SPIO were injected in the left rear limb (a), 500.000 R1H cells labeled with 50 *μ*g SPIO in the right rear limb (b). The injection volume was 50 *μ*l for both, a and b. B: Cross-section of a rat pelvis. The MR magnitude signal is displayed in grayscale, the *Fe*_2_ *O*_3_ concentration of labeled cells is color-coded. The colorbar applies to A and B.

The SVM (Fig 7) detected cancer cells at the injection sites (Fig 7A). The obtained maximum concentrations of iron oxide nanoparticles within the detected tumor volumes were similar to the injected concentrations. Nanoparticle concentrations down to ∼ 75 μM were detected at the surface of the injected tumor volumes (Fig 7B). Although the injection volume in both cases was only 50*μl*, the algorithm detected 56*μl* for the injection site at the left rear limb (a) and 86*μl* for the injection site at the right rear limb (b). This was a result of the SVM-model’s ‘halo’-type overestimation (Fig 5C), caused by the nanoparticle’s influence on the atoms’ spin, which is not limited to the voxels the nanoparticles located in. The effect on the injection site (b) is higher because the R1H cells internalized more SPIO particles during the labeling (R1H: 50*μg* in total; CC531: 30*μg* in total).

## Discussion

**Fig 8:**
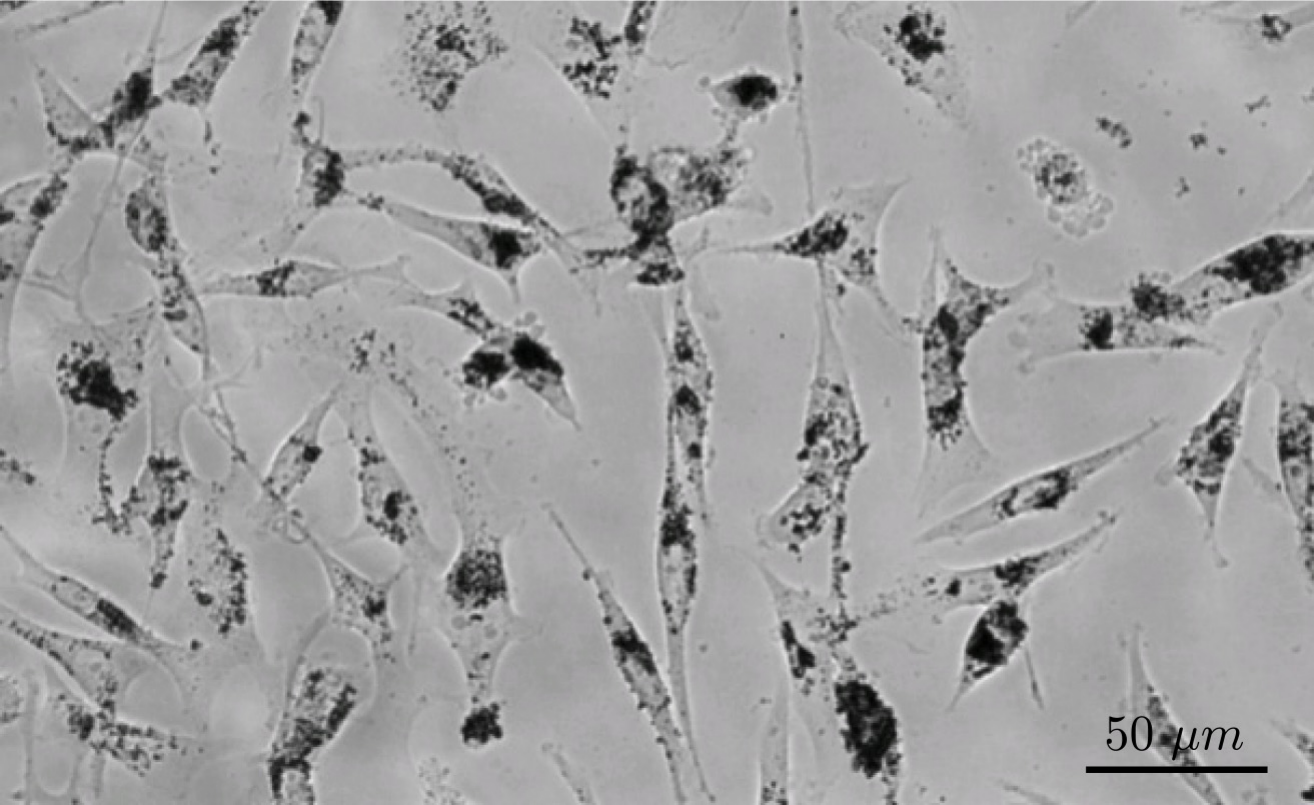
Bright-field image of MDA-MB-231 cells in culture with internalized iron oxide nanoparticles.

The current study presents a method for quantitative imaging of superparamagnetic nanoparticles in animal tissue. The study exceeds the specificity and sensitivity of previous MRI methods for non-invasive estimation of the location and concentration of iron oxide labelled cells (Fig 8). Our proof-of-concept experiments in the *in-vivo* rat model (Fig 7) suggest that our method is suitable for high precision migration and invasion studies over prolonged periods of time.

### Magnetic Particles and Cell Labeling

The SVM’s classification does not provide a cell concentration estimation, but only a segmentation of voxels presenting features characteristic for nanoparticles. Hence, the cell concentrations in each inlay (Table 1), respectively the cell numbers stated as the detection limit, are only valid for cells with ∼90 pg *Fe*_2_ *O*_3_ per cell and only shortly after the labelingg (before cell division). The SVM, however, classifies the voxels of the MRI dataset (nearly) independently from their nanoparticle concentration, and therefore is applicable to different superparamagnetic labelings and nanoparticle concentrations. Cell-accumulations with intracellular iron contents between 10 and up to 100 pg per cell which have been used in previous studies [5, 12], can be reliably localized. For a calibration of the concentration-estimation, a phantom containing different iron oxide concentrations needs to be measured in advance.

### *In-vitro* studies

Because superparamagnetic particles act as a strong enhancer of proton spin dephasing, the magnitude and phase data of voxels in the vicinity of superparamagnetic nanoparticles are affected. The nanoparticles’ influence can be quantified with features extracted from the magnitude data (Fig 2) and phase data (Fig 3). The interpretation of these features lead to a dataset consisting of voxels with two classifications: *containing labeled cells* and *not containing labeled cells* (Fig 4A). Because the ${\text{R}}_{2}^{*}$ relaxation rate is a direct measurement for the concentration of iron oxide nanoparticles, linking the positive classified voxels with the absolute value of the relaxation rate gives a map showing the locations of labeled cells and the concentrations of the intracellular nanoparticles (Fig 4B and 4C). The detection limit of the training dataset was 30 cells per mm^3^ (≈19 *μ*M *Fe*_2_ *O*_3_), giving a theoretical limit of 2 cells per voxel (0.4*mm*)^3^ (Fig 5A). This detection limit translates into an iron content of ∼180 pg per voxel. Whereas the localization algorithm detected the complete 19 *μ*M inlay in the training dataset, it was only able to find approximately 40% of the 19 μM inlet in the evaluation datasets (Fig 5B and 5D and Table 1).

The sensitivity for the SPIO containing inlays in the evaluation phantom was 0.83 ± 0.26 (mean ± std) on average (Table 2). Excluding the lowest concentration (19 μM Fe_2_O_3_) resulted in a mean sensitivity of 0.95 ± 0.05 (mean ± std). The SVM’s specificity however, increases with decreasing SPIO concentration (Table 2). Therefore, when the SVM classifies a voxel with a low iron oxide concentration as *containing labeled cells*, the probability to be a *true positive* is ≈ 1.

### *In-vivo* studies

The SVM-based localization method and the *in-vitro* pre-trained model were also used to extract iron oxide labeled tumor cells from a dataset covering the rat’s pelvis area with two injections of labeled cells in the rear limbs (Fig 6). Both of the 50 *μl* injections (left rear limb: 500,000 CC531 cells labeled with 30 μg, right rear limb: 500,000 R1H cells labeled with 50 μg) were correctly classified, but their volume was slightly overestimated. The maximum concentrations were determined correctly, however (Fig 7). The specificity achieved in the *in-vitro* experiments is confirmed by the *in-vivo* results: the SVM model is able to distinguish the iron oxide nanoparticles from air, bones and tissues with low proton density. Therefore, our method is able to unambiguously identify and locate SPIO labeled cells of two different tumor cell lines in the described *in-vivo* rat model (Fig 7B).

### Post-Processing

The ‘halo’-like overestimation of the compartments with labeled cells (Figs 5C and 7B) leaves room for improved feature extraction algorithms. Since the localization algorithm is designed in a modular way, new features can easily be added to the SVM training and prediction processes. However, since the localization method is supposed to be a supportive system for visual evaluation of the cancer cells’ migration behavior *in-vivo*, a classification generating false positives is preferred to a model that is too selective and, therefore, tends to ignore voxels containing labeled cells.

## Conclusion

The proposed method combines superparamagnetic nanoparticles, multiparametric MRI, and support vector machine based post-processing. It provides qualitative and quantitative maps of iron oxide particle distributions with a high specificity and sensitivity. Consequently, the proposed methodology is valuable for *in-vivo* monitoring of the migration and invasion of iron oxide labeled cells non-invasively and repeatedly over prolonged periods of time.

## Materials and Methods

### Cells

MDA-MB-231 human breast carcinoma cells (ATCC, LGC Standards) were used for *in-vitro* studies, and R1H rat rhabdomyosarcoma cells (gift from Dr. Andreas Brandl, Department of Plastic and Hand Surgery University of Erlangen, Erlangen, Germany) or CC531 rat colon carcinoma cells (gift from Dr. Andreas Brandl) were used for *in-vivo* studies.

MDA-MB-231 cells were grown in Dulbecco’s modified Eagles medium (DMEM) with 10% fetal bovine serum (FBS), 2mM L-glutamine, and 1g glucose. R1H cells were grown in DMEM with 10% FBS 2mM L-glutamine and 4.5g glucose. CC531 cells were grown in Roswell Park Memorial Institute medium (RPMI) 1640 with 20% FBS and 2mM L-glutamine.

Cultures were passaged with trypsin/EDTA digestion, reseeded in 35mm culture dishes at 200,000 cells per well and grown for two days until confluent.

### Magnetic Particles

Iron oxide nanoparticles have previously been shown to be non-toxic, bio-inert up to concentrations of 100 μg μl^−1^ [4, 6, 10] and to act as a strong enhancer of proton spin dephasing [10, 13]. We use 17 nm iron oxide (maghemite) nanoparticles (Sigma) as a contrast agent. To facilitate particle uptake by the cells, the particles were coated with poly-L-lysine (PLL) by incubating them overnight in a 0.01% (w/v) PLL solution [10, 14].

### Cell Labeling

Unless noted otherwise, 50 *μg* of nanoparticles were added to confluent cells grown in a 35 mm plastic cell culture dish (≈ 1 *μg* *Fe*_2_ *O*_3_ per 10,000 cells), and incubated for 24 h. Intracellular iron oxide content was 90 pg per cell on average (Fig 8). Intracellular SPIO clusters are split up between daughter cells during proliferation with an average ratio of 0.85 : 0.15 [10]. Over the time course of 14 days, the cells successively degrade the iron oxide particles into free paramagnetic iron ions [10]. To minimize the effects of particle partitioning during cell division and particle degradation over time, experiments were conducted directly after cell labeling.

### *In-vitro* studies

Agarose imaging block phantoms (each w × h × l 3.8 cm × 2.3 cm × 8.3 cm) containing 5 compartments with labeled cells were used to train and evaluate the machine learning based localization algorithm. The cylindrical compartments (Ø × h 1 cm × 0.8 cm) consisted of a 2:1 dilution series of 2% agarose and labeled cells. The highest concentration was 480 labeled cells per mm^3^ (≈ 313 μM *Fe*_2_ *O*_3_), the lowest concentration was 30 cells per mm^3^ (≈ 19 μM *Fe*_2_ *O*_3_). The compartments were evenly distributed along the main axis of the phantoms. An agarose imaging phantom with identical compartments but without iron oxide particle compartments served as baseline control (*ground truth*).

### *In-vivo* studies

To evaluate the localization algorithm *in-vivo*, labeled cells at different concentrations were injected into the left and right rear limbs of Sprague Dawley rats. The animals were cared for in accordance with the standards of the Committee on the Ethics of Animal Experiments of the Friedrich-Alexander-University Erlangen-Nuremberg and under a protocol approved by the local district government (Regierung von Mittelfranken). Prior to MRI, rats were anesthetized with isofluorane (5% for induction and 1.5% for maintenance of anesthesia). As a reference (*baseline*), an MRI scan of the animal was performed before injection with labeled cells. Afterwards, 500,000 CC531 cells labeled with 30 μg of Fe_2_O_3_ nanoparticles were suspended in 50 μl DMEM and injected into the left rear limb. 500,000 R1H cells labeled with 50 μg of Fe_2_O_3_ nanoparticles, also suspended in 50 μl DMEM, were injected into the right rear limb. Subsequent MRI scans were then performed at various time points between 18 hours and 24 days after labeling. At the end of the study, animals were sacrificed with CO_2_.

### Magnetic Resonance Imaging

MRI was performed on a 4.7 T BRUKER BioSpec scanner (Ettlingen, Germany) with a free bore of 40 cm, equipped with a BG-A 20S gradient system (gradient amplitude: 200 $\frac{\text{mT}}{m}$). The volumes of interest were positioned in the scanner’s isocenter. Magnitude and phase data were acquired using a multiparametric 3D FLASH gradient-echo pulse sequences with different echo times (TE = 2.54 ms, 3.78 ms, 11.34 ms), a repetition time TR = 200 ms, and a flip angle of 25°. The *in-vitro* data were acquired using 2 averages, an imaging matrix of 128 × 256 × 60 pixel and a field of view of 4.99 × 9.98 × 2.4 cm. Scan-time was 5.4 h in total. The *in-vivo* data were acquired without averaging using an imaging matrix of 192 × 192 × 128 pxl and a field of view of 7.49 × 7.49 × 5.12 cm. Scan-time for all three sequences was 4.1 h in total. The spatial resolution for all scans was 0.39 × 0.39 × 0.40 cm. For the agarose phantoms, multiple measurements were performed on different days.

### Post-Processing

Raw data were reconstructed to magnitude and phase image data in the DICOM format on-site and converted to the ANALYZE format. Unless noted otherwise, the subsequent post-processing was performed with algorithms written in MATLAB. The complete dataflow from the image acquisition to the iron oxide concentration map indicating labeled cells is schematically illustrated in Fig 1. The post-processing was performed with the full 3D data set, although the figures show only representative 2D images.

#### Feature extraction

The following features were extracted from the magnitude data (Fig 2):

- Signal intensity (*ρ*): For each voxel of the dataset the signal intensity was extracted from the complex image data: $\rho \left(x,y,z\right)=\text{abs}(\hat{\rho }\left(x,y,z\right))$. The signal intensity was calculated for all TE separately.
- Volume of interest (VOI): A binary map of the magnitude dataset from the TE = 2.54 ms sequence was calculated based on spatial fuzzy clustering with automated thresholding [15] to select regions of the dataset for further processing by the SVM.
- Hyper-/hypo-intensities (Δ*ρ*): Since the nanoparticles act as a contrast agent their presence results in a change of the signal intensities ($\rho^{{\text{TE}}_{i}}$) compared to the *baseline* ($\rho_{\text{baseline}}^{{\text{TE}}_{i}}$) $\Delta \rho^{{\text{TE}}_{i}}=\text{abs}\left(\rho^{{\text{TE}}_{i}}-\rho_{\text{baseline}}^{{\text{TE}}_{i}}\right)$. $\Delta \rho^{{\text{TE}}_{i}}$ is calculated voxel-wise after image registration (see below) for each TE separately.
- Spin-spin relaxation rate (${\text{R}}_{2}^{*}$): Based on the iron oxide’s proton spin dephasing, the signal intensities decay faster in areas where the nanoparticle concentrations are present than in areas without nanoparticles. (${\text{R}}_{2}^{*}$) was calculated for each voxel from an exponential fit to the decay of the signal intensities at the three different echo times [10, 16]. The iron oxide concentration was then computed from the (${\text{R}}_{2}^{*}$) as described in [10]. For determining the relationship between (${\text{R}}_{2}^{*}$) and iron oxide concentration, the nanoparticle-containing inlays of the agarose block phantoms were used.

The proton spin dephasing due to the presence of iron oxide particles affects not only the magnitude data but even stronger the often neglected phase data. Therefore, the following features were extracted from the phase data (Fig 3):

- Wrapped phase values (*ϕ*): For each voxel of the dataset, the phase was calculated: $\phi \left(x,y,z\right)\equiv \text{Arg}\;\hat{\rho }\left(x,y,z\right)={\text{tan}}^{-1}\;(\text{Im}\;\hat{\rho }\left(x,y,z\right)/\text{Re}\;\hat{\rho }\left(x,y,z\right))$. The wrapped phase dataset was computed for each TE separately.
- Unwrapped phase values (*φ*): Since the wrapped phase contains values only within [*π*, *π*) and therefore shows phase-jumps, the phase needs to be unwrapped to obtain the absolute phase values. Unwrapping was performed in 3D with the standard best-pair-first region merging algorithm [17].
- Unreliable phase values (unrel.*φ*): The quality of the phase data is prone to artifacts due to high noise levels and partial volume effects. By extrapolating the phase values of the three different echo times to the phase at the time of the excitation pulse, unreliable voxels can be identified and eliminated according to previous studies [18].
- Short range perturbations (s): The presence of SPIO nanoparticles has a high influence on the surrounding phase values. By applying a band-pass filter in the frequency domain (as described in [19, 20]), these short range perturbations caused by the particles can be detected. The particles can be localized by finding the center of mass of the volume defined by the phase perturbations of the same congruency that surround the nanoparticles in all 3 dimensions [21]. Short range perturbations measured at the second TE (3.74 ms) gave the best results regarding noise and sensitivity.
- Perturbation propagation (p*-map*): Similar to the ${\text{R}}_{2}^{*}$ relaxation rate calculated from the magnitude, the nanoparticles show not only characteristic phase patterns in space but also in time. By analyzing the short range perturbations and their sources at multiple TEs after excitation and comparing them to the ground truth, a perturbation propagation map is computed. Because short range perturbations propagate with a higher speed away from sources with higher susceptibility, this information can be used to localize the nanoparticles.

#### Feature alignment

To compare the single feature images with the ground truth image, they have to be spatially aligned. Therefore, we register the ground truth magnitude image from the first TE to the magnitude images after SPIO particle injection, also from the first TE, using a normalized mutual information method [22]. The ground truth image is the moving template; it is deformed, or warped, until it best matches the reference (the image containing SPIO particles), and not the other way round to avoid interpolation and partial volume effects within the datasets containing the labeled cells. First, a rigid registration is performed for affine alignment followed by a non-rigid registration [22]. The rigid registration matrix and the deformation field of the non-rigid magnitude registration are saved and applied to the corresponding phase datasets.

The sensitivity and specificity of the nanoparticle localization algorithm described in this study depends on the correct combination of all features. For example, voxels with a low proton density but without iron oxide contrast agents show low signal intensity and modest ${\text{R}}_{2}^{*}$ rate, similar to voxels containing iron oxide [8]. However, voxels with iron oxide also display a characteristic phase behavior that is absent in native tissue.

#### Feature classification

To detect the presence of iron oxide from a characteristic pattern of multiple features, support vector machines (SVM) [23] were used. Training of the SVM was performed on a sub-set (15% of the voxels) of the data from the MRI scans of the agarose block phantom. We manually classified each randomly selected voxel of the training data as *containing* or *not containing* iron oxide, based on our a-priori knowledge of the location of the nanoparticles inlays in the phantom block. For automatic classification, we used a C-Support Vector Classification (C-SVC) with a radial basis function kernel (*k*(***x***_***i***_, ***x***_***j***_) = *exp*(−*γ*||***x***_***i***_ − ***x***_***j***_||^2^)). Here, *x*_*i*_ and *x*_*j*_ are a set of features *x* at different spatial location *i* and *j* of the MRI image, and *γ* is a ‘sharpness’ prefactor that was optimized for our application. The SVM classifies the feature combination *x* at each voxel location *i* as *containing* or *not containing* iron oxide. This is done by solving an optimization problem with regularization on the training dataset, whereby the error between the SVM prediction (containing or not containing iron oxide) and the manual classification is minimized. The regularization parameter and the sharpness prefactor *γ* were self-optimized using the so-called five-fold cross validation method [23]. The pre-trained model was then applied to all subsequent MRI scans. After SVM classification, a map with the ${\text{R}}_{2}^{*}$ rates for each voxel classified as *containing nanoparticles* is generated to estimate the local iron oxide concentration as described in previous studies [10].

#### Sensitivity and specificity

The SVM’s sensitivity and specificity of the training phantom was compared to the evaluation phantom. Therefore, we quantified *true positives* ($\hat{=}$ voxels that are correctly classified as *containing labeled cells*), *false positives* ($\hat{=}$ voxels that are falsely classified as *containing labeled cells*), *true negatives* ($\hat{=}$ voxels that are correctly classified as *not containing labeled cells*), and *false negatives* ($\hat{=}$ voxels that are falsely classified as *not containing labeled cells*) The sensitivity, the algorithm’s ability to identify positive results, was calculated by *sensitivity* = *number of true positives*/*number of real positives*. The specificity, the algorithm’s ability to identify negative results, was calculated by *specificity* = *number of true negatives*/*number of real negatives*.
